# Monokaryotic *Pleurotus sapidus* Strains with Intraspecific Variability of an Alkene Cleaving DyP-Type Peroxidase Activity as a Result of Gene Mutation and Differential Gene Expression

**DOI:** 10.3390/ijms22031363

**Published:** 2021-01-29

**Authors:** Nina-Katharina Krahe, Ralf G. Berger, Martin Witt, Holger Zorn, Alejandra B. Omarini, Franziska Ersoy

**Affiliations:** 1 Institut für Lebensmittelchemie, Gottfried Wilhelm Leibniz Universität Hannover, Callinstr. 5, 30167 Hannover, Germany; rg.berger@lci.uni-hannover.de (R.G.B.); franziska.ersoy@lci.uni-hannover.de (F.E.); 2 Institute of Technical Chemistry, Gottfried Wilhelm Leibniz University Hannover, Callinstr. 5, 30167 Hannover, Germany; witt@iftc.uni-hannover.de; 3 Institute of Food Chemistry and Food Biotechnology, Justus Liebig University Giessen, Heinrich-Buff-Ring 17, 35392 Giessen, Germany; holger.zorn@uni-giessen.de (H.Z.); Alejandra.B.Omarini@lcb.chemie.uni-giessen.de (A.B.O.); 4 INCITAP Institute of Earth and Environmental Sciences of La Pampa (CONICET-UNLPam) National Scientific and Technical Research Council-National University of La Pampa, Mendoza 109, Santa Rosa CP 6300, La Pampa, Argentina

**Keywords:** alkene cleavage, basidiomycota, biocatalysis, dikaryon, dye-decolorizing peroxidase (DyP), gene expression, gene mutation, intraspecific variability, monokaryon, *Pleurotus sapidus*

## Abstract

The basidiomycete *Pleurotus sapidus* produced a dye-decolorizing peroxidase (PsaPOX) with alkene cleavage activity, implying potential as a biocatalyst for the fragrance and flavor industry. To increase the activity, a daughter-generation of 101 basidiospore-derived monokaryons (MK) was used. After a pre-selection according to the growth rate, the activity analysis revealed a stable intraspecific variability of the strains regarding peroxidase and alkene cleavage activity of PsaPOX. Ten monokaryons reached activities up to 2.6-fold higher than the dikaryon, with MK16 showing the highest activity. Analysis of the *PsaPOX* gene identified three different enzyme variants. These were co-responsible for the observed differences in activities between strains as verified by heterologous expression in *Komagataella phaffii*. The mutation S371H in enzyme variant PsaPOX_high caused an activity increase alongside a higher protein stability, while the eleven mutations in variant PsaPOX_low resulted in an activity decrease, which was partially based on a shift of the pH optimum from 3.5 to 3.0. Transcriptional analysis revealed the increased expression of PsaPOX in MK16 as reason for the higher PsaPOX activity in comparison to other strains producing the same PsaPOX variant. Thus, different expression profiles, as well as enzyme variants, were identified as crucial factors for the intraspecific variability of the PsaPOX activity in the monokaryons.

## 1. Introduction

Many small aromatic aldehydes and ketones are known for their olfactory properties and are of high interest for the fragrance and flavor industries [1]. Their synthesis via chemical oxidative cleavage of the corresponding alkenes is possible [2,3], but a rising demand for natural products and sustainable production processes favors alternative strategies, such as biocatalysis [4,5]. The bottleneck of these bioprocesses is sufficient enzyme activity [6]. To improve their yields, different genetic engineering techniques are available [7], but are refused by the general public and classified as not suitable for “natural and safe processes” (European Directive 2001/18/EC) [4,8]. Thus, alternative strategies are needed.

*Basidiomycota*, which belong to the higher fungi (*Dikarya*), pass through different life cycle phases [9]. In the dikaryotic mycelium (dikaryon, DK), two independent nuclei coexist throughout vegetative growth. During fructification, basidia are formed, in which karyogamy (fusion of the nuclei) occurs. Meiosis and the generation of four uninucleate basidiospores follow. Hereby, variability of the progenies will result from genetic recombination [9,10]. Germination of the basidiospores causes the formation of monokaryotic mycelia (monokaryon, MK, haploid), which may later fuse to a new dikaryon (plasmogamy), if they are mating-compatible, and, thereby, reinitiate the life cycle [9]. In consequence of the genetic and resulting phenotypic diversity of the basidiospore-derived monokaryons, the basidiomycetous life cycle offers a new option to improve enzyme activities and other traits without the use of genetic engineering.

Several studies demonstrated that monokaryons differ in mycelial growth rate [11,12,13,14,15] and enzyme activities [8,11,12,14,16]. The latter were improved in selected monokaryotic progenies in comparison to the parental dikaryon [12,14]. However, few studies deal with the elucidation of the biochemical reasons for this diversity [12,17]. Castanera et al. suggested that a higher laccase activity observed for the dikaryon of *Pleurotus ostreatus* in comparison to its parental monokaryons was due to non-additive transcriptional increase in *lacc6* and *lacc10* [17]. In contrast, Linke et al. found a higher carotene degrading activity of a *P. ostreatus* monokaryon as a result of a higher secreted activity of Lacc10 [12]. The specific reasons remained unknown, because mutations and differences in gene expression were experimentally excluded. Heterozygosity in genes was proposed as reason for intraspecific variability, as in studies by Eichlerová and Homolka [11] and del Vecchio et al. [8], but never experimentally verified.

**Figure 1:**
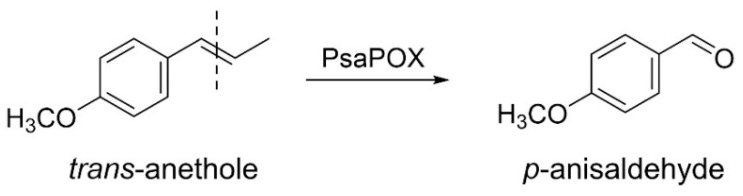
Alkene cleavage of *trans*-anethole into *p*-anisaldehyde by the dye-decolorizing peroxidase PsaPOX.

While some studies deal with the intraspecific diversity of monokaryons regarding laccase and other lignolytic activities [8,11,12,13,16,17], to the best of our knowledge, no study exists that deals with a dye-decolorizing peroxidase (DyP) activity. DyPs (EC: 1.11.19) form a new superfamily of heme peroxidases that differ highly from other classes of heme peroxidases regarding their amino acid sequence, protein structure, and catalytic residues [18]. A GXXGD-motif, which contains the catalytic aspartic acid, as well as a ferrodoxin-like fold, is typical for these enzymes [18,19]. In addition to the distal (above the heme plane) catalytic aspartic acid, which is replaced by a histidine in other heme peroxidases, a distal arginine and a proximal histidine are located in the active site (heme pocket) [18,20]. There, reductive heterolytic cleavage of hydrogen peroxide and substrate oxidation take place [20]. In contrast, oxidation of bulky substrates was proposed to occur at a surface-exposed oxidation site containing a tryptophan residue involving a long-range electron transfer [21]. Furthermore, a surface-exposed Mn^2+^-oxidation site was detected for two DyPs [22,23]. DyPs are known to oxidize different dyes, especially xenobiotic anthraquinone dyes, as well as classical peroxidase substrates, such as ABTS (2,2’-azino-bis(3-ethylbenzthiazoline-6-sulphonic acid)) and phenolic compounds [19,24,25]. However, only a recently identified DyP (PsaPOX) has been found to cleave aryl alkenes, such as (*E*)-methyl isoeugenole, *α*-methylstyrene, and *trans*-anethole (Figure 1) [23]. The resultant aldehydes are odor-active volatiles used in the fragrance and flavor industry [1]; therefore, PsaPOX is a potential biocatalyst for flavor production.

The objective of the present study was to utilize the intraspecific variability of monokaryotic progenies to improve the alkene cleavage activity of *P. sapidus*. A further purpose was the elucidation of the origin of the diversity of enzyme activity. To the best of our knowledge, this is the first report presenting intraspecific diversity concerning a DyP-type peroxidase for monokaryons and their corresponding dikaryon and, in addition, the first study identifying intraspecific differences of monokaryons concerning enzyme activities as result of a gene mutation.

## 2. Results and Discussion

### 2.1. Analysis of Monokaryons

#### 2.1.1. Pre-Selection of Monokaryons by Analysis of the Radial Growth Rate

**Figure 2:**
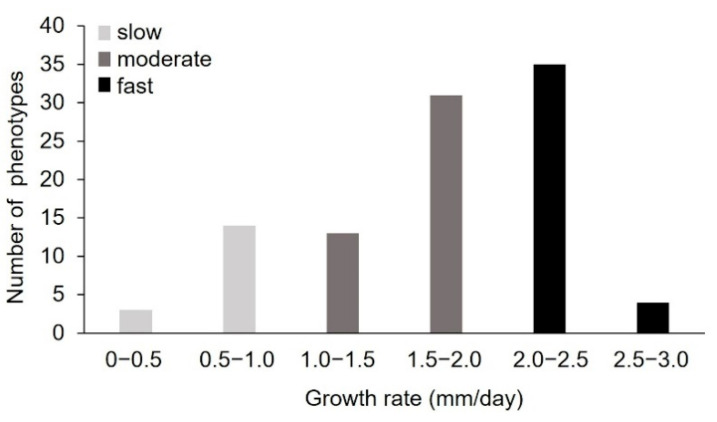
Distribution of vegetative growth rate of the *P. sapidus* monokaryons grown on standard nutrient liquid (SNL) agar plates. The strains were categorized as slow-, moderate-, and fast-growing strains according to the growth rate.

To generate new *P. sapidus* strains with an increased alkene cleavage activity, spores were collected from the basidiocarp after fructification of the dikaryotic strain. The colonies derived from the basidiospores were microscopically analyzed to confirm the monokaryotic state through the absence of clamp connections [26]. To assess the physiological diversity of the strains, the growth rate of 101 monokaryotic isolates on standard nutrient liquid (SNL) agar plates was determined as a polygenetic and easy-to-measure trait (Figure 2) [13,14,15,17,27]. The measured growth rates ranged from 0.24 ± 0.05 to 2.55 ± 0.08 mm/day, with most strains (66%) exhibiting rates between 1.5 and 2.5 mm/day. According to the growth rate, the *P. sapidus* strains were categorized into slow- (0–1.0 mm/day, 17% of the isolates), moderate- (1.0–2.0 mm/day, 45%), and fast- (2.0–3.0 mm/day, 39%) growing isolates. The dikaryon showed a growth rate of 2.26 ± 0.11 mm/day and was thus classified as fast-growing.

The intraspecific variability of the growth rate, which has also been shown for other basidiomycota [12,13,14,15,28], is an indicator for the biochemical diversity within the *P. sapidus* population and led to the assumption that the strains will also differ in other traits. Thus, five slow- (MK16, MK21, MK23, MK64, MK66), four moderate- (MK34, MK42, MK49, MK101), and five fast-growing (MK5, MK13, MK75, MK84, MK93) representative monokaryons (MK), as well as the parental dikaryon (DK), were selected for further analysis.

#### 2.1.2. Profiling of Alkene Cleavage and Peroxidase Activity in Monokaryons

**Figure 3:**
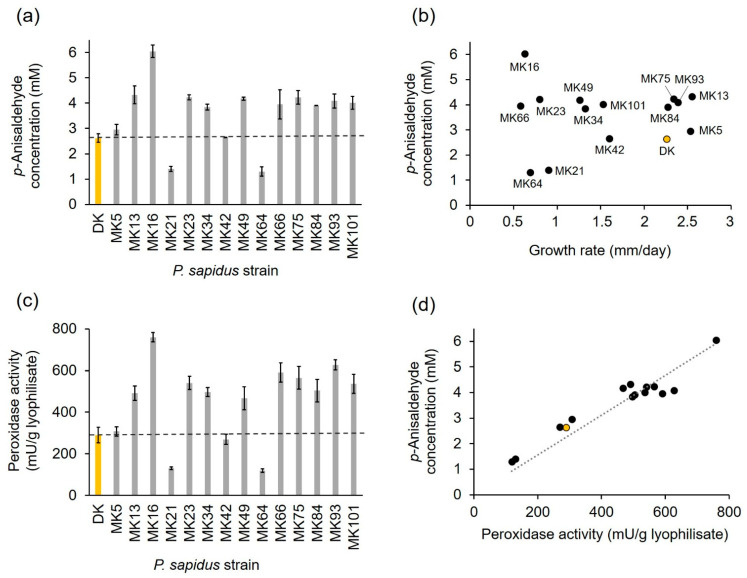
Alkene cleavage activity in comparison to peroxidase activity and growth rate of selected monokaryons and the parental dikaryon (yellow bar or circle) of *P. sapidus*. (**a**) Enzymatically generated concentration of *p*-anisaldehyde after conversion of *trans*-anethole (33.5 mM) using 30 mg/mL finely ground lyophilized *P. sapidus* mycelium in the presence of 25 mM MnSO_4_, 100 µM H_2_O_2_, and 50 mM sodium acetate buffer pH 3.5 for 16 h at RT and 200 rpm. (**b**) Comparison of alkene cleavage activity for the lyophilized *P. sapidus* mycelium after submerged cultivation and radial growth rate for the cultivation on SNL agar plates. (**c**) Peroxidase activity of the lyophilized mycelium. (**d**) Comparison of alkene cleavage and peroxidase activity of the lyophilized mycelium of the *P. sapidus* strains. *p*-Anisaldehyde concentrations are the average of duplicate experiments, and peroxidase activities the average of triplicate experiments of two independent biological replicates (with standard deviations shown as error bars in (**a**,**b**)). Growth rates are the average of two independent biological replicates cultivated under the same conditions.

After submerged cultivation, the lyophilized mycelium of the fourteen pre-selected monokaryons and the parental dikaryon was used for the biotransformation of *trans*-anethole to *p*-anisaldehyde to analyze the alkene cleavage activity of the strains (Figure 3a). The parental dikaryon produced 2.62 mM *p*-anisaldehyde, which was similar to the product concentrations obtained for MK5 and MK42, while MK21 and MK64 generated an approximately two-fold lower *p*-anisaldehyde concentration. All other monokaryons exhibited higher alkene cleavage activities (1.5–1.7-fold) than the dikaryon, while the activity of MK16 reached a 2.5-fold value. According to their activity, and in comparison, to the results for the dikaryon, the strains were categorized into four groups: (I) low active strains (MK21 and MK64); (II) moderately active strains (DK, MK5, and MK42); (III) highly active strains (MK13, MK23, MK34, MK49, MK75, MK84, MK93, MK101); and (IV) very highly active strains (MK16).

The results evinced that the analyzed *P. sapidus* strains varied not only in the growth rate, but also in the alkene cleavage activity (Figure 3b). No correlation between growth and alkene cleavage activity was detected (Figure 3b; coefficient of determination *R*^2^ = 0.12). This finding was consistent with other studies, which found no correlation between colony appearance or growth and enzyme activity [11,12,14].

The cleavage of *trans*-anethole into *p*-anisaldehyde by the parental *P. sapidus* strain was the result of the activity of the DyP-type peroxidase PsaPOX [23]; therefore, the peroxidase activity of the monokaryons was determined in addition to the alkene cleavage activity. As expected, the peroxidase activity also varied between the strains (Figure 3c) and correlated well with the ability to cleave *trans*-anethole (Figure 3d, coefficient of determination *R*^2^ = 0.82).

Classification of the strains according to their peroxidase activity resulted in the same four activity groups as defined for the alkene cleavage activity (I: ~two-fold lower than DK; II: similar to DK; III: 1.6–2.0-fold higher than DK; and IV: 2.6-fold higher than DK), which each contained the same strains as described above. Thus, it was shown that the different peroxidase and alkene cleavage activities were most likely evoked by variations of PsaPOX resulting from genetic variations between the fungal strains caused by the sexual reproductive cycle [9,10]. However, the precise reason for the different phenotypes remained to be elucidated. Possible factors included enzyme mutations, different gene expression rates, or differences on the regulative level, such as posttranscriptional gene silencing, different transposable elements contents, or factors influencing protein stability and degradation [17,29,30,31,32,33].

The present study revealed not only intraspecific variability concerning growth, peroxidase, and alkene cleavage activity, but also some improvement of these enzyme activities (alkene cleavage activity: 1.5–2.5-fold; peroxidase activity: 1.6–2.6-fold) for most of the analyzed monokaryons, especially MK16, in comparison to the parental dikaryon (Figure 3a,c). Thus, the opportunity to generate and identify optimized strains by natural reproduction and selection of the resulting progenies was verified. This is consistent with results of previous studies [11,12,14].

#### 2.1.3. Phenotypic Stability of Alkene Cleavage Activity in Sequential Cultivations

**Figure 4:**
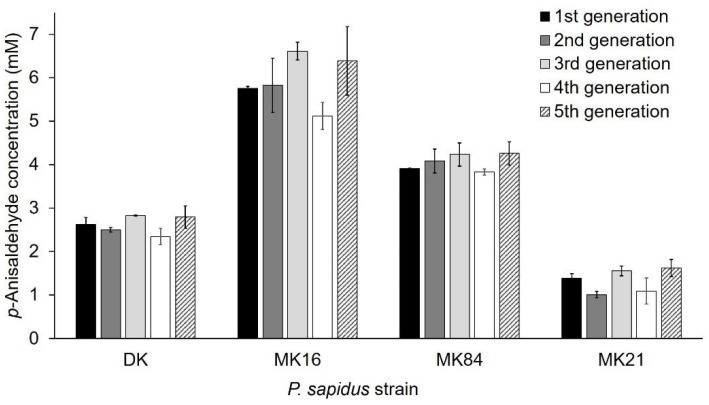
Stability of alkene cleavage activity towards *trans*-anethole of selected monokaryons and the parental dikaryon over five serial generations. Values are the average of duplicate experiments of two independent biological replicates with standard deviations shown as error bars.

Phenotypic instability of dikaryons during long-term cultivation resulting in altered properties, such as the growth rate, is known in the literature and is caused by genetic exchanges and recombination between the nuclei of the dikaryon [34]. In contrast, the growth rate of haploid monokaryons of *Schizophyllum commune* [34] and laccase and carotene degrading activity of monokaryotic *P. ostreatus* strains [12] were stable over serial propagation for several months. However, only a few studies concerning the long-term stability [12,34,35], especially regarding the production of enzymes [12] in monokaryons and dikaryons, were published. For this reason, the phenotypic stability regarding alkene cleavage activity was exemplarily examined with *trans*-anethole for the parental dikaryon (activity group II, see Section 2.1.2) and three monokaryons (activity group I (MK21), III (MK84), IV (MK16)) over five sequential generations. Sub-cultivation to the fifth generation did not influence the ability to cleave *trans*-anethole within any of the strains (Figure 4, variance among the replicate lines was between 4–18%). Thus, the observed variability between the strains was stable. This is the second study that proved the stability of an enzyme activity for basidiomycetous monokaryons and the parental dikaryon during serial propagation over several months [12].

#### 2.1.4. Comparison of PsaPOX from Selected *P. sapidus* Strains

As mentioned above, the observed stable intraspecific variabilities of the analyzed *P. sapidus* strains regarding peroxidase and alkene cleavage activity were attributed to differences concerning PsaPOX. To confirm this, the PsaPOX mRNA sequence of representative monokaryons of each activity group (see Section 2.1.2; I: MK21 and MK64; II: MK5 and MK42; III: MK75, MK84, and MK101; IV: MK16) was reverse transcribed, amplified, and sequenced. An alignment (Figure S1, Supplementary Materials) revealed four different DNA sequences: sequence 1 [23] identified for the dikaryon and MK5, sequence 2 identified for MK42 (differing in 2 bp to sequence 1), sequence 3 identified for MK21 and MK64 (differing in 65 bp to sequence 1), and sequence 4 identified for MK16, MK75, MK84, and MK101 (differing in 4 bp to sequence 1). Even though two different genotypes (gDNA level, corresponding to sequence 1 and 3, plus introns) were detected for the dikaryon (Figure S1, Supplementary Materials), no sequence in addition to sequence 1 was identified for the dikaryon via clone sequencing on the mRNA level. This indicated monoallelic expression, which was previously described for another gene of *P. ostreatus*, a near relative of *P. sapidus* [36]. Sequence 3 originated from the unexpressed gene variant; therefore, sequence 2 and 4 should have represented the consensus sequence of the expressed and unexpressed dikaryotic gene variant if they resulted solely from recombination events during meiosis. The genotype at position 36 in sequence 2 and 4 was in accordance with the genotype of the unexpressed dikaryotic gene sequence (sequence 3), while the main part of sequence 2 and 4 showed a higher identity to sequence 1 (Figure S1, Supplementary Materials). Thus, the genotype at position 36 is probably the result of meiotic recombination. In contrast, the genotypes at positions 468 (sequence 2 and 4), 1111, and 1112 (sequence 4) must have resulted from random mutations, because they did not correspond to either of the dikaryotic genotypes at the respective positions (see Figure S1, Supplementary Materials). The high contribution of sequence 4 (present in four of eight tested strains) indicates that the mutations occurred at the early stage of fructification so that the new sequence was distributed over multiple cell divisions before formation of the basidia. Thus, the mutated sequence would have been present in several of the generated spores originating from different basidia, resulting in multiple monokaryons with sequence 4.

**Figure 5:**
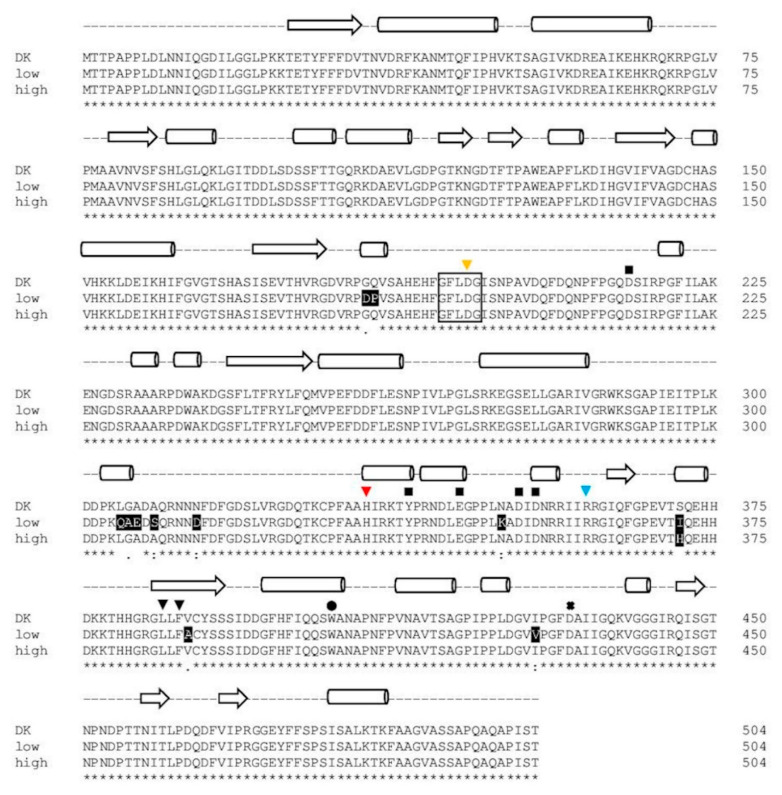
Alignment of the amino acid sequence of the PsaPOX variants. DK: PsaPOX_DK; low: PsaPOX_low; high: PsaPOX_high. Inverted triangles show amino acids important for heme binding (H334 (red) functions as ligands for heme and the four other amino acid residues form a hydrogen peroxide binding pocket). Aspartic acid (D433), which forms a hydrogen bond with histidine to stabilize compound I (oxidized heme after transfer of two electrons to H_2_O_2_), is indicated by a cross. The black box indicates the GXXDG motif containing the catalytic aspartic acid residue (D196, yellow inverted triangle), which cleaves H_2_O_2_ heterolytically with the help of the neighboring arginine (R360, blue inverted triangle) to form compound I, and the circle presents an exposed tryptophan (W405) potentially involved in a long-range electron transfer. Important amino acids for Mn^2+^-oxidation (D215, Y339, E345, D352, and D354) are marked by squares. Secondary structure elements are shown above the alignment (arrow: *β*-sheets, barrel: *α*-helices, dashed line: random coil) and were predicted by the SWISS-MODEL server [37] using the X-ray crystal structure of *Pleos*-DyP4 from *P. ostreatus* (PDB-ID 6fsk). Asterisks indicate conserved residues, colons indicate equivalent residues, and dots indicate partial residue conservations. Observed mutations compared to the parental sequence (DK) were highlighted in black with white font. The alignment was performed with Clustal Omega (European Bioinformatics Institute, Hinxton, UK) [38].

While sequence 1 and 2 resulted in identical protein sequences (PsaPOX_DK: parental sequence identified for the dikaryon), the protein resulting from sequence 3 (PsaPOX_low) carried eleven mutations (G184D, Q185P, L305Q, G306A, A307E, A309S, N314D, N350K, S371I, V388A, I429V), while the protein resulting from sequence 4 (PsaPOX_high) carried one amino acid mutation (S371H) (Figure 5). None of the observed mutations resulted in the exchange of an amino acid known to be important for DyP activity (see Figure 5). However, PsaPOX_low seemed to be correlated with a lower enzyme activity than that exhibited by the dikaryon and PsaPOX_high with a higher activity, as all representative low active strains (MK21 and MK64) contained PsaPOX_low and all highly active strains (MK16, MK75, MK84, MK101) contained PsaPOX_high. This was in line with the finding that MK5 and MK42 contained PsaPOX_DK as well as an activity similar to the dikaryon. Linke et al. [12] examined the influence of a sequence mutation as the possible reason for a higher activity of Lacc10 in *P. ostreatus* MK51 in comparison to the dikaryon as well other monokaryons, but did not find any correlation. To the best of our knowledge, no study has yet been able to attribute phenotypic variability of mono- and dikaryons concerning enzyme activity to a specific gene mutation.

### 2.2. Analysis of the Recombinant PsaPOX Variants

#### 2.2.1. Activity of the Recombinant PsaPOX Variants

To verify the influence of the detected amino acid sequence exchanges on the PsaPOX activity, the sequences 1, 3, and 4 coding for the three PsaPOX variants (PsaPOX_DK, PsaPOX_low, and PsaPOX_high, respectively, see Section 2.1.4) were heterologously expressed in *K. phaffii* as previously described for the parental sequence (sequence 1) [23]. After 72 h of cultivation, the best performing colonies produced maximal peroxidase activities of up to 142 U/L for PsaPOX_DK [23], 138 U/L for PsaPOX_low, and 86 U/L for PsaPOX_high. The recombinant PsaPOX variants were purified by Ni-NTA affinity, which resulted in comparable electrophoretic purities of the enzymes (Figure S2, Supplementary Materials). Two protein bands were detected, which corresponded to the unmodified peroxidase (lower band, calculated molecular mass using ExPASy [39]: 54.9 kDa) and the *N*-glycosylated enzyme (upper band, result of posttranslational modifications by *K. phaffii*) [23]. The percentage of unmodified (19–21%) and glycosylated (79–81%) enzyme was similar, as shown by an evaluation of the protein band intensities. This was supported by the results of a bioinformatic analysis of the PsaPOX sequences, which identified the same three putative *N*-glycosylation sites (N42, N81, and N458) for all enzyme variants. Thus, an influence of different glycosylation patterns on the enzyme activity or solubility was excluded.

**Figure 6:**
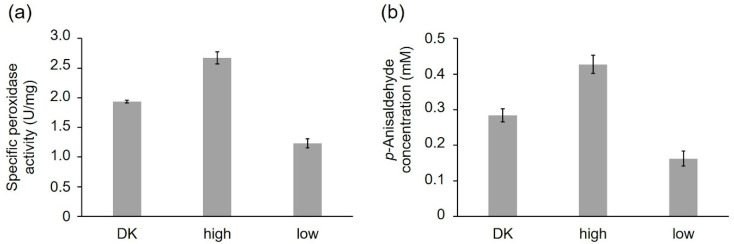
Comparison of the PsaPOX variants regarding their peroxidase and alkene cleavage activity. (**a**) Specific peroxidase activity. Values are the average of triplicate measurements using three independent replicates with standard deviations shown as error bars (**b**) *p*-Anisaldehyde concentration after bioconversion of *trans*-anethole (6.7 mM) using 0.25 mg/mL PsaPOX variant in the presence of 25 mM MnSO_4_, 100 µM H_2_O_2_, and 50 mM sodium acetate buffer pH 3.5 for 16 h at RT and 200 rpm. Values are the average of duplicate independent experiments with standard deviations shown as error bars. DK: PsaPOX_DK; high: PsaPOX_high; low: PsaPOX_low.

The purified peroxidase variants were compared, regarding their specific peroxidase and alkene cleavage activity using identical protein concentrations (peroxidase activity: 1 ng/mL; alkene cleavage activity: 0.25 mg/mL) (Figure 6a,b). PsaPOX_DK reached a specific peroxidase activity of 1.94 ± 0.02 U/mg, while PsaPOX_low showed a specific activity of 1.23 ± 0.07 U/mg and PsaPOX_high of 2.67 ± 0.10 U/mg (Figure 6a). Similar results were obtained for the alkene cleavage activity, which was represented by the produced *p*-anisaldehyde concentration after biotransformation of *trans*-anethole by the PsaPOX variants. In the presence of PsaPOX_DK, 0.28 ± 0.02 mM *p*-anisaldehyde was produced, whereas PsaPOX_low generated 0.16 ± 0.03 mM and PsaPOX_high 0.43 ± 0.02 mM product (Figure 6b).

The results verified that the observed point-mutations (see Figure 5) in the sequences of PsaPOX_low and PsaPOX_high caused a lower and higher activity, respectively, and was the reason for the higher or lower activity of the (very) highly (activity group III and IV) or low active (activity group I) *P. sapidus* strains (see Section 2.1.2). Thus, this is the first experimental proof that a gene mutation resulted in different activities of monokaryons and the parental dikaryon, although an activity change due to amino acid exchanges of a given enzyme in general is well documented [21,40,41].

However, the ratio of activity difference between the recombinant PsaPOX_DK and PsaPOX_low (peroxidase activity: 1.6-fold, alkene cleavage activity: 1.7-fold) or PsaPOX_high (peroxidase activity: 1.4-fold, alkene cleavage activity: 1.5-fold) was slightly less distinctive than for the parental *P. sapidus* dikaryon in comparison to the low (peroxidase activity and alkene cleavage activity: 2-fold) or (very) highly active (peroxidase activity: 1.6–2.6-fold, alkene cleavage activity: 1.5–2.5-fold) strains (Figure 3a,c and Figure 6a,b). This indicated that further factors, such as different levels of expression (see Section 2.3) were involved in the observed phenotypic diversity of the *P. sapidus* strains. MK16 especially (classified as very highly active, activity group IV, see Section 2.1.2), reached an at least 1.3- (peroxidase activity) to 1.4-fold (alkene cleavage activity) higher activity than all other analyzed *P. sapidus* strains, although it contained the PsaPOX_high variant like the highly active strains.

#### 2.2.2. Structural Analysis of the PsaPOX Variants

**Figure 7:**
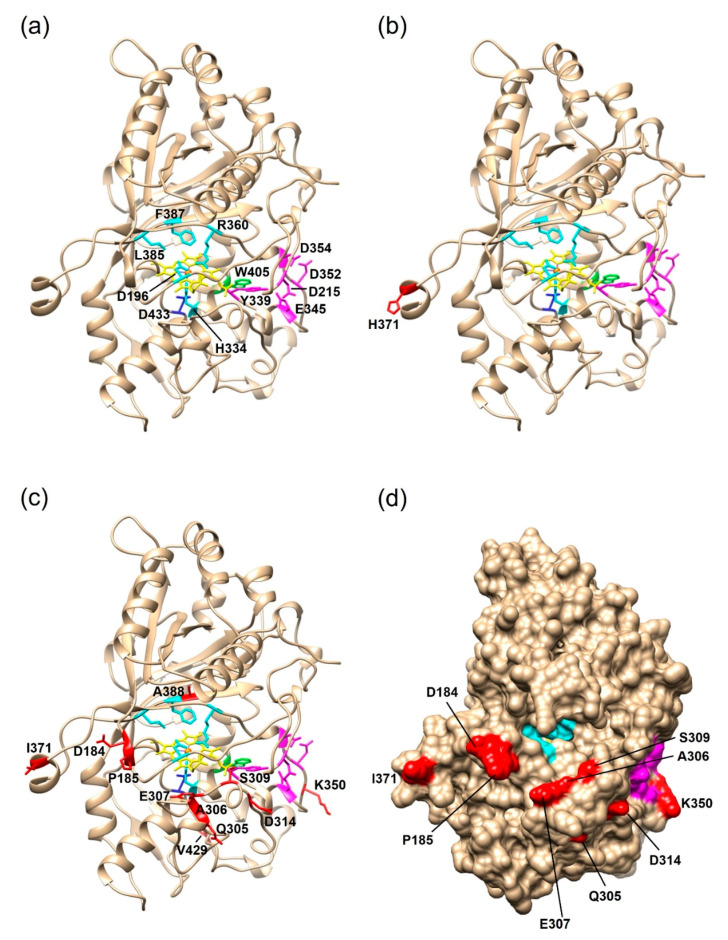
Structural homology models of the three PsaPOX variants. The models were generated with the SWISS-MODEL server using the X-ray crystal structure of *Pleos*-DyP4 (PDB-ID 6fsk). (**a**) Overall fold of PsaPOX_DK showing the typical ferredoxin-like fold with the heme cofactor (shown in yellow, the heme iron is highlighted in orange) sandwiched between distal and proximal sides. Conserved amino acids of the active site/hydrogen peroxide pocket (proximal H334, distal D196, R360, L385, and F387) are shown in cyan. D433, which forms a hydrogen bond with H334 to stabilize compound I (oxidized heme after transfer of two electrons to H_2_O_2_), is presented in blue, and the exposed W405 potentially involved in a long-range electron transfer is shown in green. Important amino acids for Mn^2+^-oxidation (D215, Y339, E345, D352, and D354) are highlighted in magenta. Overall fold of (**b**) PsaPOX_high, and (**c**) PsaPOX_low. Mutations present in the enzyme variants are shown in red. (**d**) Solvent access surface of PsaPOX_low, showing the heme active site in cyan, the Mn^2+^-oxidation site in magenta, and the position of the surface-exposed amino acid exchanges in red.

To identify the positions of the amino acid mutations and assess a possible impact on the enzyme activity, structural homology models of the PsaPOX variants were generated on the SWISS-MODEL server [37] (Figure 7). The template was the X-ray crystal structure of the *Pleos*-DyP4 from *P. ostreatus* (PDB-ID 6fsk; 94% identity). The S371H mutation of PsaPOX_high was surface-exposed, and at least 23 Å away from the conserved amino acids of the active site (cf. Figure 7a,b). Thus, it seemed highly unlikely that the amino acid exchange directly influenced the catalytic activity, which disagreed with the observed activity improvement for the PsaPOX_high variant. However, some studies reported an activity increase by distant mutations [42,43,44]. For example, the mutation (I238Y) of a vanillyl alcohol oxidase located at a distance of 33 Å from the flavine–adenine dinucleotide in the active site cavity increased the turnover number for creosol, even though structural changes, which could account for the improvement, were not identified by X-ray crystallography [43]. The structural homology models used in this study cannot serve to analyze structural changes. In addition, conformational differences due to the fact that another protein was used as template cannot be excluded. It is possible that the loop region containing S371H was further orientated towards the entrance of the active site as a consequence of the flexible random coil (Figure 7b), so that S371H may affect and improve the accessibility to the catalytic site. Another hypothesis was that S371H influenced the folding or unfolding of the peroxidase, which resulted in a higher percentage of correctly folded proteins or an increase in protein stability (compare with Section 2.2.3). The influence of single-point mutations on the folding and stability of enzymes has been reported before [45].

The eleven amino acid exchanges in PsaPOX_low were mostly positioned at the surface of the enzyme (Figure 7c,d). However, one mutation (V388A) was located in the heme cavity at distances of 6, 7, and 10 Å to the conserved residues F387, R360, and D196 of the hydrogen binding pocket, respectively (Figure 7c). The exchange of valine to the smaller alanine may have caused a conformational change in the hydrogen binding pocket, which could explain the lower activity of the *P. sapidus* strains containing the PsaPOX_low enzyme. Furthermore, most of the detected mutations (G184D, Q185P, L305Q, G306A, A307E, A309S, N314D, N350K, and S371I) led to a polarity change, which may have resulted in a rearrangement of the three-dimensional protein structure and therefore to a lower protein stability and activity. The change of enzyme activity and stability by conformational changes is known from the literature [40,46,47]. Another hypothesis was that the substrate access to the heme cavity was complicated by several mutations (G184D, Q185P, L305Q, G306A, A307E, and A309S), which mostly increased the steric hindrance near the substrate entrance (Figure 7d), as assumed in the case of two other DyP-type peroxidases [40,41]. Furthermore, a negative charge was added by the exchange of glycine to aspartic acid at position 184 (pI of Asp: 2.98 [48]; p*K*_a_ of Asp: 3.7 [49]), which probably aggravated the accommodation of the anionic substrate ABTS. Interestingly, position 371, which was mutated in the PsaPOX_high variant (S371H), was also mutated in PsaPOX_low (S371I). This indicated that the position is important for the activity of PsaPOX, despite its remote location in the predicted protein structure model (Figure 7b,c). X-ray crystallography will be necessary to ascertain the actual protein folding at this position. Point-mutation studies could be performed to ascertain the influence of position 371 on the enzyme activity as part of a follow-up study.

#### 2.2.3. Comparative Biochemical Characterization of the PsaPOX Variants

**Figure 8:**
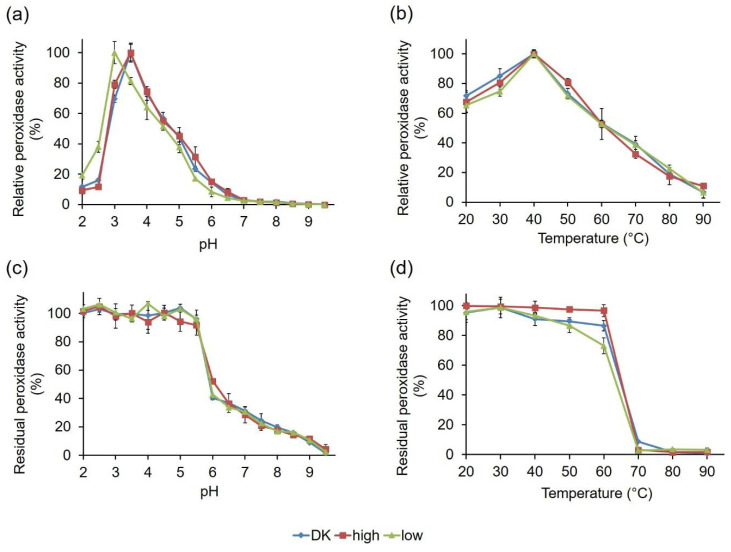
Influence of pH and temperature on activity and stability of the PsaPOX variants. (**a**) pH optimum and (**b**) temperature optimum. Relative peroxidase activity was defined as the percentage of activity detected with respect to the highest activity of the corresponding enzyme variant in each experiment. pH stability (**c**) was determined after incubation of PsaPOX in Britton–Robinson buffer, ranging from pH 2.0 to 9.5 for 1 h at RT, and temperature stability (**d**) was determined after incubation at 20–90 °C and pH 3.5 for 1 h. Residual activities were determined at pH 3.5 and 40 °C. Values are the average of triplicate experiments with standard deviations shown as error bars. DK: PsaPOX_DK; high: PsaPOX_high; low: PsaPOX_low.

To check if the observed activity variations of the PsaPOX variants were associated with differing biochemical characteristics, the influence of pH and temperature on PsaPOX activity and stability was determined using ABTS in the presence of hydrogen peroxide as a substrate (Figure 8). PsaPOX_DK, which has been characterized for its biochemical properties before [23], as well as PsaPOX_high, showed pH optimums of 3.5 (Figure 8a). In contrast, the optimum of PsaPOX_low was shifted to the more acidic range at pH 3.0. This shift was probably caused by several mutations in the primary sequence of PsaPOX_low (Figure 5 and Figure 7c), which led to polarity changes at different sites (G184D, Q185P, L305Q, G306A, A307E, A309S, N314D, N350K, and S371I), including insertion of acidic and basic amino acids (G184D, A307E, N314D, N350K). Thus, it was assumed that PsaPOX_low underwent conformational changes, which resulted in the shift of the pH optimum [40]. A second hypothesis is that the amino acid exchange G184D near the entrance of the active site complicated the accommodation of the anionic substrate ABTS, due to an additional negative charge at pH 3.5 (pI of Asp: 2.98 [48]; p*K*_a_ of Asp: 3.7 [49]) [40]. At pH 3.0, this was most likely prevented because the carboxyl side chain of 184D was protonated and the amino acid was uncharged. PsaPOX_low reached only 80% activity at pH 3.5, which was used for all other assays analyzing the specific peroxidase and alkene cleavage activity (Section 2.2.1), while PsaPOX_DK exhibited 100% activity (Figure 8a). It was concluded that the lower activity of PsaPOX_low in comparison to PsaPOX_DK was partly caused by the shifted pH optimum. However, the measured peroxidase activity (1.23 U/mg, Figure 6a) was lower than that calculated for pH 3.5 (1.55 U/mg, equal to 80% of activity of PsaPOX_DK). Further factors seemed to be involved.

All three PsaPOX variants showed a similar pH stability profile, with the highest stability between pH 2.0 and 5.5 (residual peroxidase activity ≥90% after 1 h of incubation) (Figure 8c). At pH values higher than 6.0, the stability decreased drastically, which was most likely caused by a reduced solubility and changes of the protein structure, which may have resulted in protein aggregation [23]. At pH 6.0, PsaPOX_high exhibited a residual activity of 52%, which was slightly higher than for PsaPOX_DK (40%) and PsaPOX_low (42%). Thus, S371H may have resulted in a small stability increase, as assumed above (see Section 2.2.2).

Determination of the influence of temperature on the peroxidase activity showed that the PsaPOX variants possessed the same optimum (40 °C, Figure 8b). Furthermore, they shared a similar temperature–activity profile. On the contrary, differences for the temperature stability, which was determined after an incubation at different temperatures for 1 h, were detected (Figure 8d). While >96% peroxidase activity remained in the range between 20 and 60 °C for PsaPOX_high, the residual activity of PsaPOX_DK decreased between 40 and 60 °C from 89 to 84%. PsaPOX_low exhibited similar residual activities as PsaPOX_DK between 20 and 50 °C, but at 60 °C the residual activity (72%) was 12% and 24% lower than for PsaPOX_DK and PsaPOX_high, respectively. At higher temperatures (≥70 °C), a high loss of activity occurred for all PsaPOX variants due to protein aggregation [23], resulting in activities ≤5%. PsaPOX_high exhibited a higher stability, and PsaPOX_low a lower stability, than the parental variant. Thus, S371H (PsaPOX_high) seemed to have a stabilizing effect on the tertiary structure of PsaPOX, while the amino acid exchanges in the sequence of PsaPOX_low resulted in a destabilization of the protein structure, which caused a higher sensitivity to heat-induced denaturation. Changes of the thermal stability as a result of the mutation of a certain enzyme are well known [46,47].

**Figure 9:**
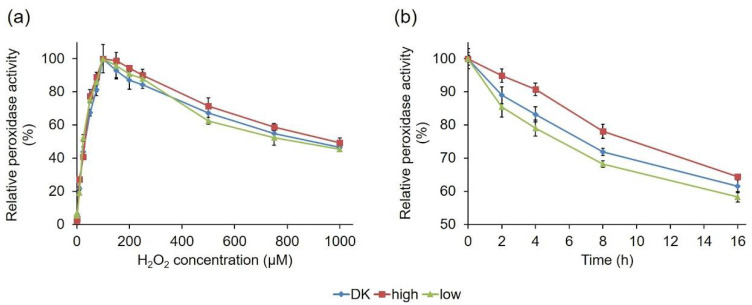
Effect of hydrogen peroxide concentration on the activity of the PsaPOX variants (**a**), and relative peroxidase activity of the PsaPOX variants during biotransformation of *trans*-anethole over 16 h (**b**). Relative peroxidase activity was defined as the percentage of activity detected normalized to the highest activity (**a**) or to the starting activity (**b**) of the corresponding enzyme variant in each experiment. Values are the average of triplicate experiments with standard deviations shown as error bars. DK: PsaPOX_DK; high: PsaPOX_high; low: PsaPOX_low.

In addition, the hydrogen peroxide dependency of the three PsaPOX variants was compared (Figure 9a). The enzymes shared a similar behavior and reached their activity optimum in the presence of 100 µM, as previously described for PsaPOX_DK [23]. In the presence of higher hydrogen peroxide concentrations, a continuous activity decrease was observed as result of suicide inhibition. This was also observed for other DyPs [50,51] and most likely the result of the formation of an inactive oxidative state of the heme in the enzyme’s active site [23,50,52,53].

PsaPOX_low and PsaPOX_high showed a changed thermal stability, and the latter also showed a slightly different pH stability in comparison to PsaPOX_DK; therefore, the stability of the PsaPOX variants during the biotransformation of *trans*-anethole over 16 h was determined (Figure 9b). In each case, an activity loss occurred during the incubation time, resulting in a remaining activity of 58% (PsaPOX_low) to 64% (PsaPOX_high) after 16 h. However, the activity loss was slower for PsaPOX_high than for PsaPOX_DK and faster for PsaPOX_low.

In conclusion, the peroxidase variants showed different stabilities under biotransformation conditions. The results were in line with the results for the thermal stability—and in the case of PsaPOX_high, also consistent with the findings for the pH stability. Therefore, they supported the deduction that the mutation in PsaPOX_high (S371H) had a stabilizing effect, while the mutations in the sequence of PsaPOX_low destabilized the peroxidase structure, thus influencing the enzyme activity.

To analyze the influence of the amino acid mutations of PsaPOX_low and PsaPOX_high on the Michaelis–Menten constant and catalytic efficiency, the corresponding kinetic parameters were calculated for the oxidation of ABTS at pH 3.5 (Table 1). The three variants showed similar Michaelis–Menten constants (25.8–33.0 µM), which were in the same range as for a DyP from *Irpex lacteus* (28 µM) [51]. Thus, no influence of the mutations on the Michaelis–Menten constant was concluded. However, the turnover numbers (*k*_cat_) and, as a consequence, the catalytic efficiencies differed (Table 1). As expected from the results for the specific activities (Figure 6a), the turnover number and the efficiency of PsaPOX_high (10.5 s^−1^ and 408 s^−1^ mM^−1^) were higher (~two-fold), while the values for PsaPOX_low (3.8 s^−1^ and 119 s^−1^ mM^−1^) were lower (~1.7-fold) than for PsaPOX_DK (6.8 s^−1^ and 206 s^−1^ mM^−1^). The results for PsaPOX_low were attributed to the changed pH optimum as well as the increased steric hindrance due to several mutations near the entrance to the active site (see Section 2.2.2), which most likely hampered the substrate access. In contrast, the higher turnover number and catalytic efficiency of PsaPOX_high was attributed to a higher percentage of correctly folded and therefore active protein due to an improved folding process or a reduced unfolding in the presence of the S371H, as assumed above (see Section 2.2.2). This would fit to the higher stability of PsaPOX_high. The comparison of the catalytic efficiencies of the PsaPOX variants with other DyP-type enzymes showed that the S371H mutation resulted not only in an improved catalytic efficiency for PsaPOX_high in comparison to the wild-type enzyme, but also in comparison to the *Pleos*-DyP4 of *P. ostreatus* (352 s^−1^ mM^−1^) [22]. However, the catalytic efficiency of a DyP from *Irpex lacteus* for ABTS (8000 s^−1^ mM^−1^) was higher [51].

### 2.3. Expression Profile of the PsaPOX Gene from Different P. sapidus Strains

**Figure 10:**
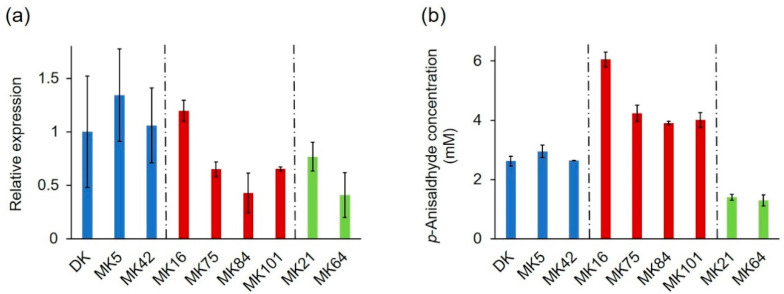
Relative expression of the *PsaPOX* gene (**a**) in comparison to the alkene cleavage activity, and (**b**) of selected monokaryons and the dikaryotic *P. sapidus* strain. Expression rates were normalized to the expression of the parental dikaryon. Values are the average of three (expression) or two (alkene cleavage activity) independent biological replicates with standard deviations shown as error bars. *P. sapidus* strains were grouped according to their PsaPOX sequence (blue: PsaPOX_DK; red: PsaPOX_high; green: PsaPOX_low).

The identified mutations of *PsaPOX*, which resulted in three differently active enzyme variants, explained most of the observed intraspecific variability of the peroxidase and alkene cleavage activity, but the residual variances indicated further underlying causes (see Section 2.2.1). At least a 1.3–1.4-fold higher peroxidase and alkene cleavage activity was detected for MK16 in comparison to all other *P. sapidus* strains, which cannot have resulted from the observed amino acid exchanges in the PsaPOX sequence. Previous studies have shown differences of the transcriptional profile of mono- and dikaryons [17,36,54,55]. Thus, a transcriptional analysis was performed to monitor the relative expression of the *PsaPOX* gene in the *P. sapidus* strains, whose coding sequence had been evaluated (Figure 10a). The strains expressed different gene variants and accordingly produced three differently active enzymes; therefore, the results were examined and categorized in respect to the present PsaPOX variant in each strain.

MK42 and the parental strain, which produced PsaPOX_DK and showed a similar peroxidase and alkene cleavage activity, also exhibited a similar expression rate (Figure 3c and Figure 10a,b). In contrast, the expression of MK5, which likewise contained the PsaPOX_DK variant, was slightly increased. This agreed with the slightly higher peroxidase and alkene cleavage activity of MK5 in comparison to the dikaryon, but the differences were smaller for the activities. This indicated the existence of unknown regulative mechanisms, such as posttranslational gene silencing (e.g., small interfering RNA and transposon-associated DNA methylation) or protein degradation [30,31,56].

*PsaPOX* expression in MK21 and MK64, both containing the PsaPOX_low variant, differed (~two-fold), even though the enzyme activities of the strains were similar (Figure 3c and Figure 10a,b). Thus, the regulation of PsaPOX_low production or degradation must have differed in MK21 and MK64.

The expression profiles of MK75, MK84, MK101, and MK16, all of which produced PsaPOX_high, were similar to the pattern of the enzyme activities (Figure 3c and Figure 10a,b). MK84 exhibited the lowest expression rate and activities; MK75 as well as MK101 demonstrated slightly higher values; while the activities and *PsaPOX* expression of MK16 was noticeable higher (≥1.8-fold). In conclusion, the higher peroxidase and alkene cleavage activity of MK16, which was classified as very highly active (see Section 2.1.2), was also the result of a higher expression of the *PsaPOX* gene. However, the difference in expression between MK16 and the other high active strains was more distinctive (≥1.8-fold) than the differences of the peroxidase and alkene cleavage activity (≥1.3-fold). The detected activities of the three strains thus have to be influenced by an additional unknown regulative factor.

So far, only two other studies [12,17] have examined the gene expression of specific genes as the reason for varying enzyme activities of monokaryons and the corresponding dikaryons. Linke et al. [12] experimentally excluded the hypothesis, while Castanera et al. [17] identified a higher laccase activity of a *P. ostreatus* dikaryon in comparison to its parental monokaryons as result of up-regulation of *lacc6* and *lacc10* expression. Previous studies dealing with the gene expression of mono- and dikaryons focused on the overall differences between these, but not on specific activities [36,54]. Thus, differences in gene expression as a factor involved in intraspecific variability of a DyP-type enzyme were confirmed for the first time.

## 3. Materials and Methods

### 3.1. Chemicals and Materials

Chemicals were obtained from Sigma-Aldrich (Seelze, Germany), Carl Roth (Karlsruhe, Germany), or Merck (Darmstadt, Germany) in p.a. quality. PCR primers were obtained from Eurofins MWG Operon (Ebersberg, Germany).

### 3.2. Fructification of P. sapidus, Isolation of Basidiospores, and Screening of Monokaryons

Culture conditions of the dikaryotic strain (*P. sapidus*, Deutsche Sammlung von Mikroorganismen und Zellkulturen, DSMZ, Braunschweig, Germany, strain no. 2866) and the procedure for the production of monosporic colonies as well as their microscopic identification have been published previously [14]. The dikaryotic strain and 101 isolated monokaryotic strains were maintained on 1.5% (*w*/*v*) SNL agar [57] and evaluated regarding their radial growth rate in duplicates. For this, 5 mm^2^ of actively growing mycelium of each strain were transferred to another SNL agar plate and incubated at 24 °C for ten days. Radial growth (increase of the colony) was measured every two days as mm/day.

### 3.3. Submerged Cultivation of P. sapidus Strains

The dikaryon (fast-growing) as well as five slow-, four moderate-, and five fast-growing monokaryotic strains were selected for submerged cultivation (in duplicate). For pre-cultivation, 1 cm^2^ of grown agar was transferred to 100 mL SNL medium and treated with an Ultraturrax homogenizer (ART Prozess- & Labortechnik, Müllheim, Germany). The pre-cultures were incubated for 5 days at 150 rpm and 24 °C. Afterwards, 6.5 g (wet biomass) of pre-grown mycelium were used to inoculate 250 mL SNL. The main culture was incubated at 150 rpm and 24 °C. After six days, the mycelium was separated from the culture supernatant by centrifugation (5000× *g*, 4 °C, 15 min) and lyophilized as described elsewhere [58]. Afterwards, the lyophilisates were finely ground.

### 3.4. Phenotype Stability of Different P. sapidus Strains

To analyze the phenotype stability of different *P. sapidus* stains regarding the *trans*-anethole cleaving activity, the dikaryotic strain as well as three monokaryons were analyzed over five generations in duplicate. For this, the strains were re-cultivated every month on a fresh SNL agar plate with the mycelia of the previous month. The mycelium of each generation was subsequently used for submerged cultivation (Section 3.3).

### 3.5. cDNA Synthesis and Gene Amplification

Isolation of total RNA from the mycelia of the *P. sapidus* strains at culture day six, cDNA synthesis, and amplification of the *PsaPOX* gene (coding sequence) were performed as described previously for the parental strain [23]. The untreated, as well as the DNase I-treated (Invitrogen, 1 U per µg RNA), RNA was used for cDNA synthesis. gDNA isolation was performed according to Aamir et al. [59] using 200 mg of mycelium (culture day six), but without RNase A treatment. The amplification of the *PsaPOX* gDNA sequence was performed as described for the coding sequence using the following cycler program: denaturation for 2 min at 98 °C, 35 cycles at 98 °C for 1 min, 62 °C for 30 s and 72 °C for 2 min, and a final elongation at 72 °C for 10 min. Analysis of PCR products, ligation, transformation in *Escherichia coli*, colony PCR, plasmid isolation, and sequencing were performed as described by Behrens et al. [24]. For sequencing of the gDNAs, the respective sequences were re-amplified from the pUC57 constructs after plasmid isolation using the standard primers M13 (5′-GTAAAACGACGGCCAGT-3′) and M13r (5′-CAGGAAACAGCTATGAC-3′) and Phusion High-Fidelity DNA Polymerase (Thermo Scientific, Wilmington, DE, USA). The thermal cycler program was as follows: denaturation for 2 min at 98 °C, 35 cycles at 98 °C for 1 min, 54 °C for 30 min and 72 °C for 2 min, and a final elongation at 72 °C for 10 min. The PCR products were purified by gel electrophoresis (1.0% agarose gels) and gel elution using the NucleoSpin^®^ Gel and PCR Clean-up Kit (Macherey-Nagel, Düren, Germany). Afterwards the PCR products were used for DNA sequencing (Seqlab, Göttingen, Germany). Translation of DNA sequences was performed using the Software SnapGene^®^ (GSL Biotech LLC, Chicago, IL, USA). Alignments were produced by ClustalOmega [38]. For the prediction of putative *N*-glycosylation sites, the NetNGlyc 1.0 Server [60] was used.

### 3.6. Heterologous Expression of the PsaPOX Variants in Komagataella phaffii

The *PsaPOX* gene variants (PsaPOX_high and PsaPOX_low) were amplified with a C-terminal 6x His tag, inserted into the *K. phaffii* pPIC9 expression vector (Invitrogen, Karlsruhe, Germany), and expressed in *K. phaffii* as described for the gene of the dikaryotic strain [23]. The *K. phaffii* transformants were grown in 500 µL YEPD (1% (*w*/*v*) yeast extract, 2% (*w*/*v*) peptone, and 2% (*w*/*v*) dextrose) in 96-well plates at 28 °C and 320 rpm for 48 h. Afterwards, the pre-cultures were transferred to the main-cultures by a medium change to 500 µL BMMY (1.34% (*w*/*v*) yeast nitrogen base, 1% (*w*/*v*) yeast extract, 2% (*w*/*v*) peptone, 100 mM potassium phosphate (pH 6), 4 × 10^−5^% (*w*/*v*) biotin, and 1% (*w*/*v*) methanol after one washing step with 500 µL sterile water. The main cultures were cultivated for 72 h at RT and 320 rpm. Gene expression was induced by daily addition of 1% (*v*/*v*) methanol.

### 3.7. His-Tag Purification of the Recombinant PsaPOX Variants

The His-tagged PsaPOX variants were purified from the *K. phaffii* culture supernatant by Ni-NTA affinity chromatography according to Nieter et al. [61]. Afterwards, the eluted fractions were analyzed by SDS-PAGE, as described elsewhere [12]. Protein band intensities were evaluated by ImageJ [62]. Protein concentrations were determined according to Bradford [63] using bovine serum albumin as standard.

### 3.8. Biotransformation

Biotransformation of 5 µL (33.5 mM) *trans*-anethole was carried out with 30 mg *P. sapidus* lyophilisate (see Section 3.3), buffered in sodium acetate (50 mM, pH 3.5) in the presence of 25 mM MnSO_4_ and 100 µM H_2_O_2_ in a total volume of 1 mL for 16 h at RT and 200 rpm according to Krahe et al. [23]. Controls (chemical blank: without lyophilisate; biological blank: with heat inactivated mycelium (2 h at 95 °C)) were performed accordingly. For the bioconversion with the recombinant peroxidase variants, 0.25 mg/mL purified recombinant protein and 1 µL (6.7 mM) *trans*-anethole were used, while all other parameters remained unchanged. Controls were performed without enzyme (chemical blank) or with heat inactivated enzyme (1 h at 95 °C, biological blank). All experiments were performed in duplicates. Sample preparation, analysis by gas chromatography, as well as quantification of the conversion product *p*-anisaldehyde was performed as described elsewhere [23]. To calculate the enzymatically generated product concentration, the product concentrations in the blanks were subtracted from the concentrations yielded with the active enzymes.

### 3.9. Peroxidase Activity

Total peroxidase activity was determined photometrically (EON™ High Performance Microplate Spectrophotometer, BioTek Instruments GmbH, Bad Friedrichshall, Germany) by monitoring the oxidation of ABTS in the presence of hydrogen peroxide at 420 nm (ε_420_ = 3.6 × 10^4^ M^−1^ cm^−1^) and 40 °C for 10 min. Samples were mixed with sodium acetate buffer (100 mM, pH 3.5), 100 µM H_2_O_2_ and 0.5 mM ABTS in a total volume of 300 µL. One unit of enzyme activity was defined as 1 µmol substrate oxidized per minute under the experimental conditions. To determine the activity of the lyophilized *P. sapidus* mycelium, the peroxidases were solubilized by pre-incubation of 30 mg lyophilized mycelium in 1 mL sodium acetate buffer (100 mM, pH 3.5) for 2 h at 4 °C and 200 rpm. Afterwards, the samples were centrifuged (5000× *g*, 4 °C, 15 min) and the supernatant was used for the ABTS assay. For the calculation of the specific peroxidase activities of the three recombinant PsaPOX variants, identical protein concentrations (1 ng/mL) of the recombinant enzymes were used. All enzyme assays were performed as triplicates. Blanks were performed with water instead of buffer and by omission of hydrogen peroxide.

### 3.10. Comparative Biochemical Characterization of the PsaPOX Variants

Effects of pH, temperature, and hydrogen peroxide concentration on peroxidase activity as well as the kinetic constants for the substrate ABTS were comparatively evaluated for the PsaPOX variants. For this, 1 ng/mL enzyme was used for the ABTS assay (Section 3.9). The pH optimum was determined using Britton–Robinson buffer [64] in a range of 2.0 to 9.5 instead of sodium acetate buffer, while for determination of the temperature optimum the peroxidase activity was examined at different temperatures (20–90 °C). For analysis of the pH stability, the enzyme variants were incubated in Britton–Robinson buffer from pH 2.0 to 9.5 for 1 h at RT before the peroxidase activity was examined at pH 3.5 (100 mM sodium acetate buffer). For determination of the temperature stability, the PsaPOX variants were incubated at different temperatures (20–90 °C) for 1 h prior to enzyme activity measurement at 40 °C. The influence of the hydrogen peroxide concentration was examined by evaluation of peroxidase activity with different H_2_O_2_ concentrations (0–1 mM). Relative activities were normalized to the highest activity and residual activities to the initial activity prior to incubation. Kinetic constants of the enzyme variants for ABTS were calculated after peroxidase measurements with varying ABTS concentration (0–300 µM) by SigmaPlot 12.5 (Systat Software Inc., Chicago, IL, USA) using nonlinear regression.

### 3.11. Quantitative Real-Time PCR

For analysis of *PsaPOX* expression, three independent biological replicates were sampled after submerged cultivation at day six of the main culture (Section 3.3). Total RNA was prepared from 300 mg frozen mycelium using the NucleoSpin^®^ RNA Plant Kit (Macherey Nagel). Nucleic acid concentrations were determined using a Nanodrop ND-1000 spectrophotometer (Thermo Scientific). RNA was treated with RNAse-free DNAse I (Invitrogen) using 1 U per µg RNA, and afterwards 1 µg total RNA was reverse-transcribed into cDNA using the FastGene Scriptase II cDNA Kit with oligo-dT primer (in 20 µL, Nippon Genetics Europe, Düren, Germany).

Quantitative real-time PCR (RT-qPCR) was performed in a CFX384 Real-Time PCR detection system (Bio-Rad Laboratories, München, Germany) using GoTaq qPCR Master Mix (Promega, Fitchburg, WI, USA). The primers qRT_*PsaPOX*_for (5′-CGATATCCTCGGaGGATTGCCA-3′) and qRT_*PsaPOX*_rev (5′-CGCTTCACGGTCCTTGACTA-3′) were chosen from homologous regions of the *PsaPOX* gene variants. Glyceraldehyd-3-phosphate dehydrogenase (*gpd3*; *gpd3*_for 5′-GCCATCAATGACCCGTTCATTG-3′ and *gpd3*_rev 5′-CCTTCTCCGCGAAGATGTGG-3′) and purine phosphorylase (*phos*; *phos*_for 3′-CATCGCAAATCATCGATCGCACC-3’ and *phos*_rev 5′-GCTCTCCAGCCATTGCACCAATT-3′), described to be the best reference genes in *P. sapidus* for the expression analysis of different peroxidase genes [65], were chosen as reference genes. The reaction mix (10 µL final volume) consisted of 5 µL GoTaq qPCR Master Mix, 0.4 µL of both primers (0.2 µM each, final concentration), 2.6 µL nuclease-free water, and 2 µL of a 1:12.5 dilution of the cDNA. The amplification program was as follows: 95 °C for 2 min, 40 cycles at 95 °C for 20 s, 64.8 °C for 30 s and 70 °C for 10 s, and a final elongation at 70 °C for 2 min. Afterwards, a melting curve analysis was performed from 70 to 95 °C with a temperature increase of 0.5 °C/0.1 s to detect potential nonspecific products. Agarose gel electrophoresis was used to check for PCR and primer specificity. Each reaction was performed in triplicate, and non-template controls were included for each primer set. Using a mixture of all sample cDNAs, PCR efficiency for each primer pair was evaluated by the dilution series method (five orders of magnitude, see Table S1, Supplementary Materials) [66]. The Bio-Rad CFX Maestro Software was used for expression data calculation using the 2^−ΔΔCT^ method [67], for which the relative quantity ΔC_T_ of the target gene was normalized with respect to the geometric mean of the relative quantities of the reference genes.

### 3.12. Sequence Accession Numbers

The nucleotide sequences of the *PsaPOX* gene variants have been deposited in the GenBank database under accession numbers MT043310 (sequence 1, parental), MT847628 (sequence 3), MT847629 (sequence 4) and MT847630 (sequence 2).

## 4. Conclusions

In this study, basidiospore-derived monokaryons of the basidiomycete *P. sapidus* were used. They showed an intraspecific variability in peroxidase and alkene cleavage activity of the DyP-type peroxidase PsaPOX. The latter was stable over five sub-cultivation cycles. Furthermore, several monokaryons, especially MK16, exhibited higher peroxidase and alkene cleavage activities than the parental dikaryon. Thus, activity improvement was achieved by natural reproduction and selection of the progenies.

Analysis of the *PsaPOX* gene revealed the existence of three PsaPOX variants in the *P. sapidus* strains, which were partly responsible for the intraspecific variability in enzyme activity, as determined by analysis of the recombinant enzymes. This is the first study that verified gene mutation as reason for a differing enzyme activity in mono- and dikaryons. PsaPOX_high contained the S371H mutation, which resulted in a higher activity as well as improved catalytic efficiency, possibly due to a measurable increased stability. However, the molecular basis for the increase in activity of PsaPOX remains to be investigated, due to the peripheral location of the mutation in the predicted protein structure. Thus, X-ray crystallography and molecular modeling to investigate protein dynamics should be performed in follow-up studies. Furthermore, protein folding studies could be useful, because the observed results may be caused by changes in protein folding or unfolding and the resulting increase in ratio of active to inactive protein. In PsaPOX_low, eleven mutations were present that led to a decreased activity as well as a reduced stability. Experiments with the single-point mutants could show which of the amino acid exchanges were crucial for the observed biochemical changes.

Transcriptional analysis of the *P. sapidus* strains identified an up-regulation of the *PsaPOX* gene expression in MK16 in comparison to the other strains containing the PsaPOX_high enzyme variant, as reason of its higher activity. In contrast, the gene expression profiles of the low active strains did not match with the observed results for the peroxidase and alkene cleavage activities. Thus, it was proposed that the strains differed in further traits, which regulated the detected PsaPOX activity.

The present study gives insights into some factors responsible for the intraspecific variability of PsaPOX activity in the monokaryotic and dikaryotic *P. sapidus* strains, but further research is needed to fully understand the regulatory processes in depth. The study identified one promising overproducing monokaryon (MK16) with potential as production strain for a commercial application of PsaPOX.

## Figures and Tables

**Table 1:** Michaelis–Menten constants (*K*_m_), catalytic constants (*k*_cat_), and catalytic efficiencies (*k*_cat_/*K*_m_) for the PsaPOX variants (1 ng/mL) using 0.5 mM ABTS as a substrate in the presence of 100 µM H_2_O_2_ and 100 mM sodium acetate buffer, pH 3.5 at 40 °C. Values are the average of triplicate experiments with indication of standard deviations. DK: PsaPOX_DK; high: PsaPOX_high; low: PsaPOX_low.

PsaPOX Variant	*K*_m_ (µM)	*k*_cat_ (s^−1^)	*k*_cat_/*K*_m_ (s^−1^ mM^−1^)
DK	33.0 ± 2.5	6.8 ± 0.1	206 ± 4
high	25.8 ± 1.6	10.5 ± 0.1	408 ± 14
low	31.9 ± 2.3	3.8 ± 0.1	119 ± 2

## Data Availability

Data Availability Statements in section.

## Supplementary Materials

The following are available online at https://www.mdpi.com/1422-0067/22/3/1363/s1, Figure S1: Alignment of *PsaPOX* reverse-transcribed mRNA sequences from different monokaryons as well as the parental dikaryon; Figure S2: SDS-PAGE analysis of the purified recombinant PsaPOX variants after Ni-IMAC; Table S1: PCR efficiency of the primer pairs used for the RT-qPCR analysis.

## Abbreviations

ABTS	2,2′-azino-bis(3-ethylbenzthiazoline-6-sulphonic acid)
DK	Dikaryon
DSMZ	Deutsche Sammlung von Mikroorganismen und Zellkulturen
DyP	Dye-decolorizing peroxidase
gpd3	Glyceraldehyd-3-phosphate dehydrogenase
MK	Monokaryon
phos	Purine phosphorylase
PsaPOX	DyP of *P. sapidus*
PsaPOX_DK	Parental PsaPOX variant
PsaPOX_high	PsaPOX variant with higher activity than PsaPOX_DK
PsaPOX_low	PsaPOX variant with lower activity than PsaPOX_DK
RT-qPCR	Quantitative real-time PCR
SNL	Standard nutrient liquid
