# Supplementary material for: Monokaryotic Pleurotus sapidus Strains with Intraspecific Variability of an Alkene Cleaving DyP-Type Peroxidase Activity as a Result of Gene Mutation and Differential Gene Expression
Source: Int J Mol Sci. 2021 Jan 29;22(3):1363. doi: 10.3390/ijms22031363 (PMC7866418; doi:10.3390/ijms22031363)
Supplement: Supplementary file 1 [file ijms-22-01363-s001.zip › Supplementary/supplementary.docx]

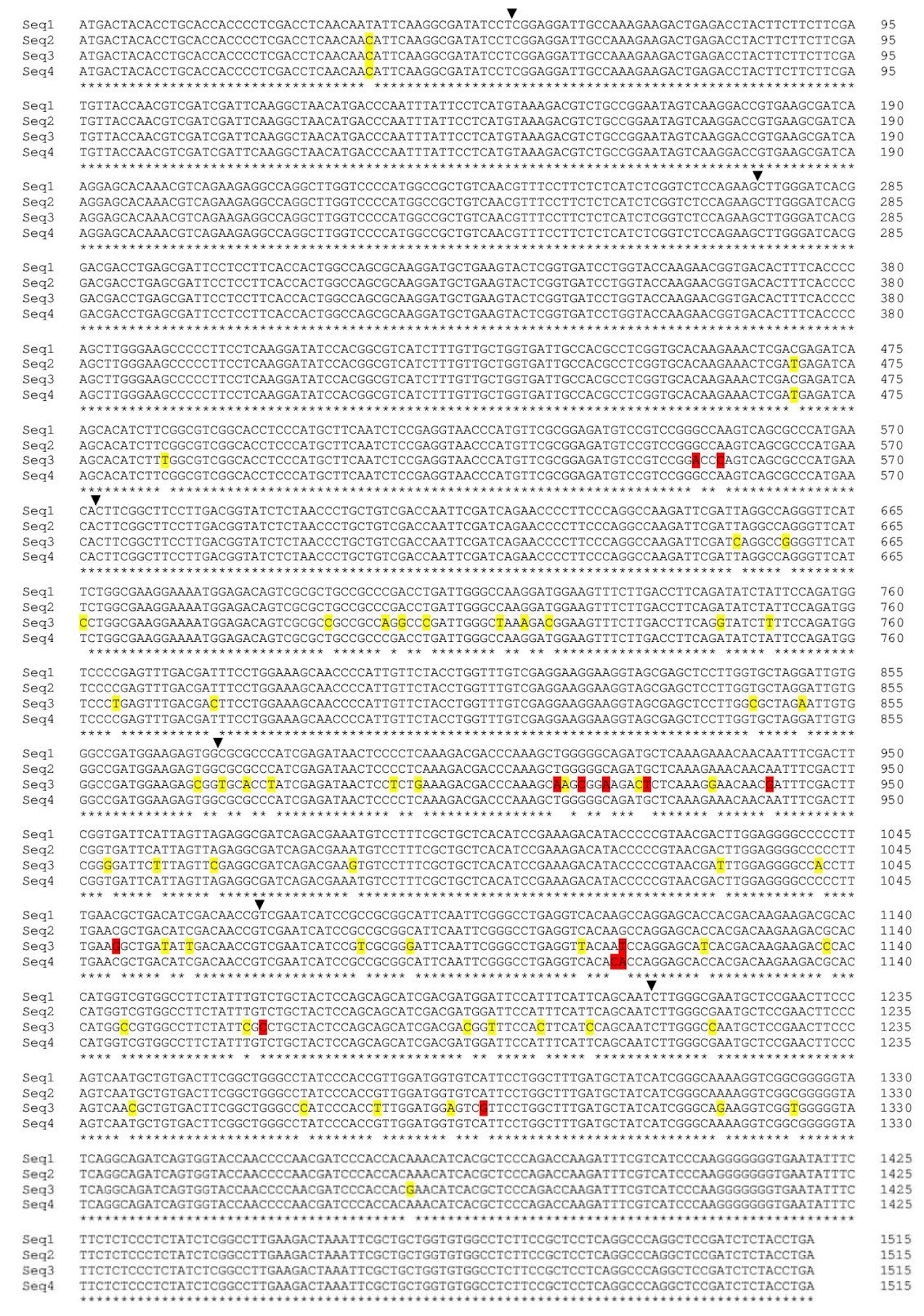


**Figure S1.** Alignment of *PsaPOX* reverse transcribed mRNA sequences from different monokaryons as well as the parental dikaryon. Seq1: sequence 1 of the dikaryon and MK5; Seq2: sequence 2 of MK42; Seq3: sequence 3 of MK21 and MK64, Seq4: sequence 4 of MK16, MK75, MK84, and MK101. Nucleotide exchanges were highlighted. Mutations resulting in amino acid exchanges are shown in red and exchanges which did not result in amino acid exchanges are presented in yellow. Alignment was performed with Clustal Omega (European Bioinformatics Institute, Hinxton, UK) [38]. Sequence 1 and 3 correspond to the coding regions of the two dikaryotic *PsaPOX* gDNA sequences. Inverted triangles indicate the position of introns.


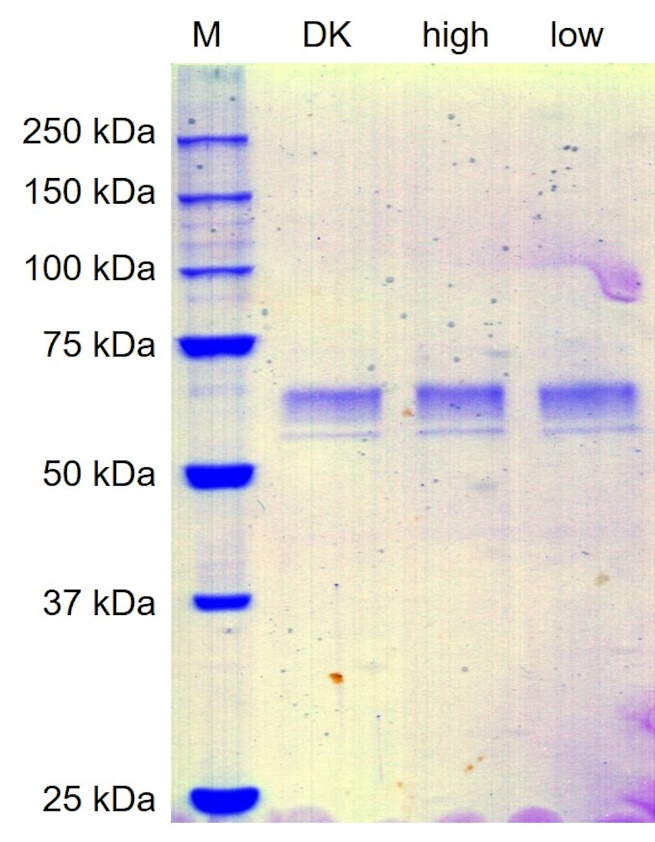


**Figure S2.** SDS-PAGE analysis of the purified recombinant PsaPOX variants after Ni-IMAC. The gel was stained with Coomassie Brilliant Blue. DK: PsaPOX_DK, high: PsaPOX_high, low: PsaPOX_low, M: molecular mass marker. Two protein bands were detected for the PsaPOX variants belonging to the unmodified (lower band) and glycosylated proteins (upper band).

**Table S1.** PCR efficiency of the primer pairs used for the RT-qPCR analysis.

| **target gene** | **primer pair** | **PCR efficiency [%]** |
| --- | --- | --- |
| *PsaPOX* | qRT_*PsaPOX*_for and qRT_*PsaPOX*_rev | 92.8 |
| *gpd3* | *gpd3*_for and *gpd3*_rev | 88.3 |
| *phos* | *phos*_for and *phos*_rev | 95.7 |
