# Supplementary figures and images for: Monokaryotic Pleurotus sapidus Strains with Intraspecific Variability of an Alkene Cleaving DyP-Type Peroxidase Activity as a Result of Gene Mutation and Differential Gene Expression
Source: Int J Mol Sci. 2021 Jan 29;22(3):1363. doi: 10.3390/ijms22031363 (PMC7866418; doi:10.3390/ijms22031363)

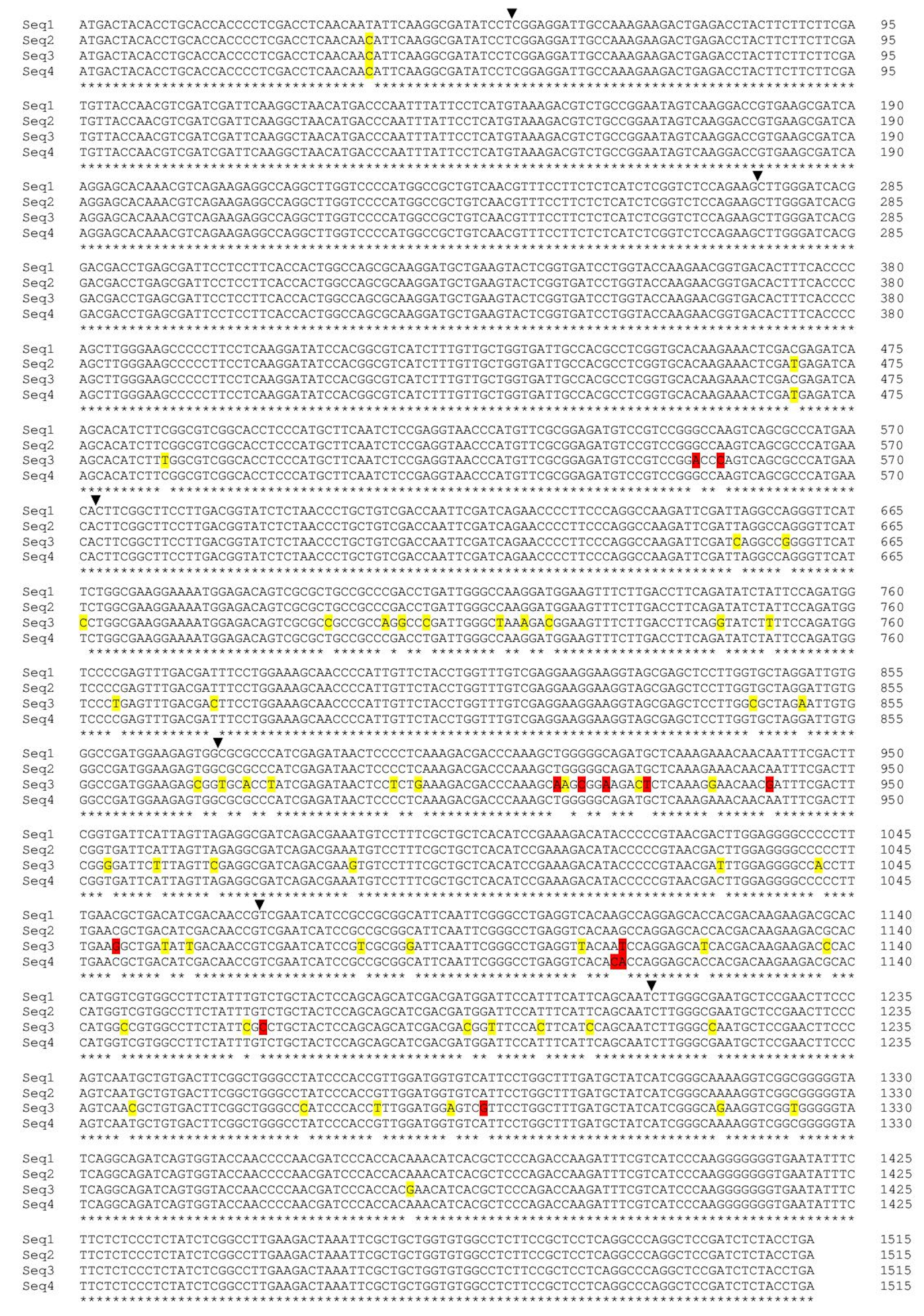

Supplement: Supplementary file 1 [file ijms-22-01363-s001.zip › Supplementary/Figure S1.jpg]

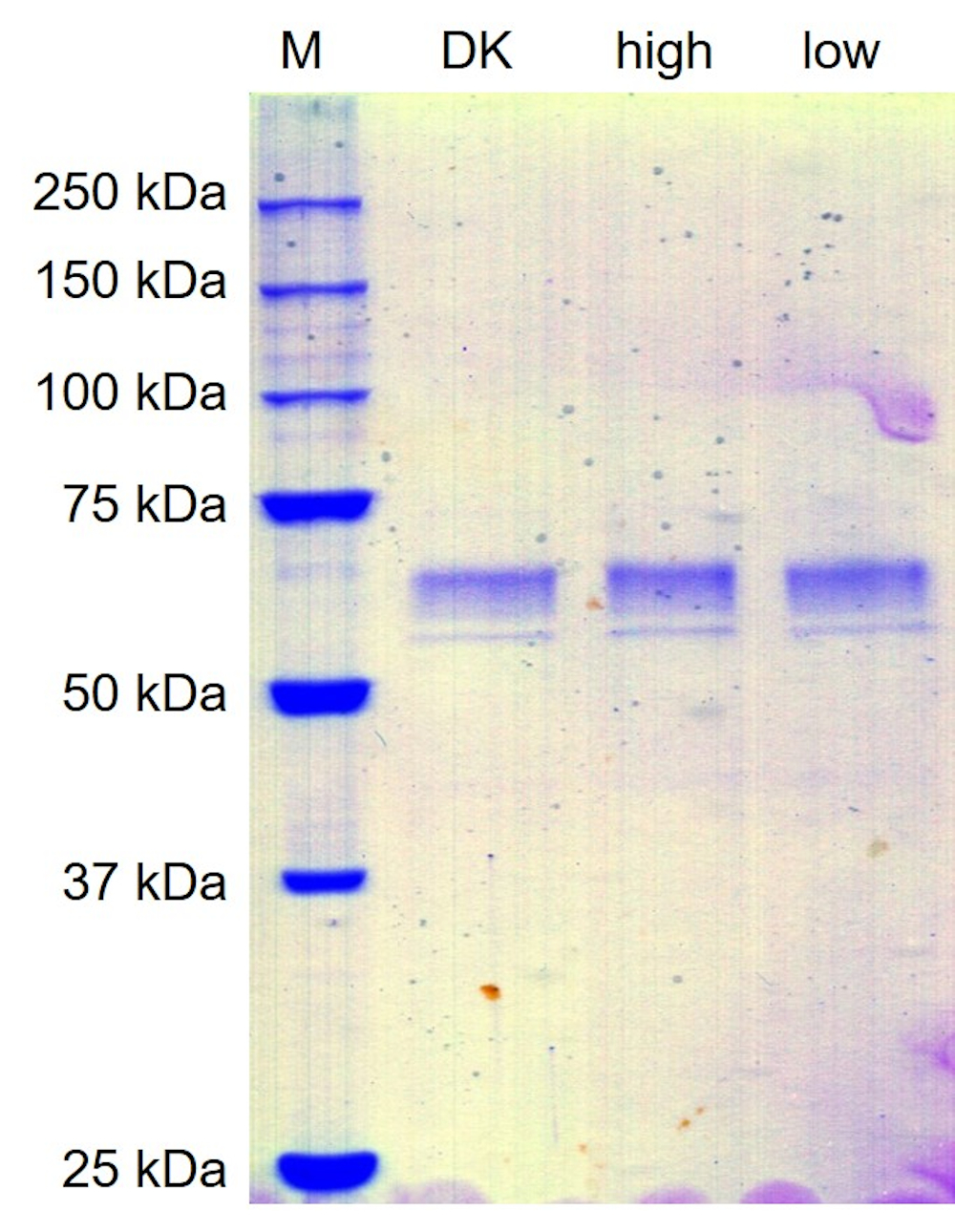

Supplement: Supplementary file 1 [file ijms-22-01363-s001.zip › Supplementary/Figure S2.jpg]
